# *Streptobacillus felis*, a member of the oropharynx microbiota of the *Felidae*, isolated from a tropical rusty-spotted cat

**DOI:** 10.1007/s10482-020-01454-x

**Published:** 2020-08-09

**Authors:** Ahmad Fawzy, Jörg Rau, Karin Riße, Nicole Schauerte, Christina Geiger, Jochen Blom, Can Imirzalioglu, Jane Falgenhauer, Alexa Bach, Christiane Herden, Tobias Eisenberg

**Affiliations:** 1 Faculty of Veterinary Medicine, Department of Medicine and Infectious Diseases, Cairo University, Cairo, Egypt; 2 Department of Veterinary Medicine, Hessian State Laboratory (LHL), Schubertstr. 60, 35392 Giessen, Germany; 3 Chemical and Veterinary Analysis Agency Stuttgart, Schaflandstr. 3/2, 70736 Fellbach, Germany; 4 Frankfurt Zoo, Bernhard-Grzimek-Allee 1, 60316 Frankfurt, Germany; 5 Bioinformatics and Systems Biology, Justus-Liebig-University Giessen, Heinrich-Buff-Ring 58, 35392 Giessen, Germany; 6 Institute for Medical Microbiology, Justus Liebig University Giessen, Schubertstr. 81, 35392 Giessen, Germany; 7 Institute of Veterinary Pathology, Justus-Liebig-University Giessen, Frankfurter Str. 96, 35392 Giessen, Germany; 8 Institute of Hygiene and Infectious Diseases of Animals, Justus-Liebig-University Giessen, 35392 Giessen, Germany

**Keywords:** *Streptobacillus felis*, Rat bite fever, Cat reservoir, Zoonosis, Immuno-histochemistry (IHC)

## Abstract

*Streptobacillus felis* is a fastidious microorganism and a novel member of the potentially zoonotic bacteria causing rat bite fever. Since its description, this is the second isolation of *S. felis* in a diseased member of the *Felidae*. Interestingly, the strain from this study was isolated from a zoo held, rusty-spotted cat (*Prionailurus rubiginosus*), with pneumonia, thereby indicating a possible broader host range in feline species. A recent preliminary sampling of domestic cats (*Felis silvestris* forma *catus*) revealed that this microorganism is common in the oropharynx, suggesting that *S. felis* is a member of their normal microbiota. Due to unawareness, fastidiousness, antibiotic sensitivity and lack of diagnostics the role of *S. felis* as a cat and human pathogen might be under-reported as with other *Streptobacillus* infections. More studies are necessary to elucidate the role of *S. felis* in domestic cats and other *Felidae* in order to better estimate its zoonotic potential.

**Electronic supplementary material:**

The online version of this article (10.1007/s10482-020-01454-x) contains supplementary material, which is available to authorized users.

## Introduction

**Fig. 1:**
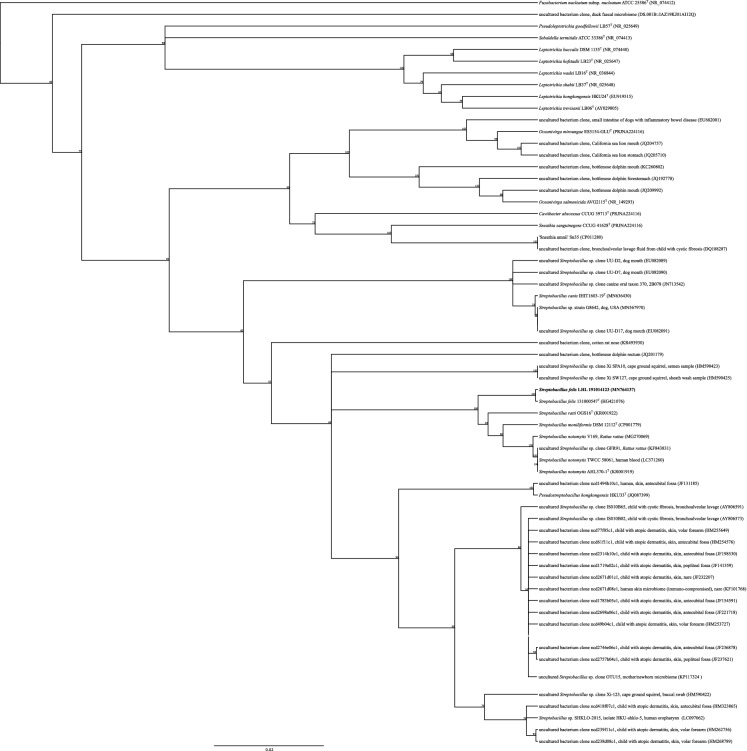
UPGMA consensus tree depicting phylotypes and species of the family *Leptotrichiaceae*. The data set was based on 16S rRNA gene sequences and processed in Geneious vers. 8.1.9 (Kearse et al. 2012) using a Clustal W nucleotide alignment with standard settings and rapid bootstrap analysis (1,000 bootstraps). GenBank accession numbers are given in parentheses. Numbers at branch nodes refer to bootstrap values; *Fusobacterium nucleatum* is used as outgroup. ^“T”^ indicating type strain; Bar, 0.02 nucleotide substitutions per site

*Streptobacillus* (*S*.) *moniliformis* (*Leptotrichiaceae, Fusobacteriales*) has been the longstanding unique species in this genus (Levaditi et al. 1925). This bacterium represents the most important causative microorganism of rat bite fever (RBF) and its food-borne variant, Haverhill fever (Eisenberg et al. 2018). RBF is typically characterized by a triad of fever, arthritis and a maculopapular, petechial or pustular rash, but severe causes of infection may include life-threatening sequelae (Eisenberg 2017; Eisenberg et al. 2017a; Gaastra et al. 2009). A number of studies have stated a risk for RBF even through contacts to various non-rodent animal species like dogs, cats, weasels and ferrets as well as livestock animals. However, the proper identification of these microorganisms was not carried out and such isolates have not been stored. Recently, [*S.*] *hongkongensis* (Woo et al. 2014), *S. felis* (Eisenberg et al. 2014), *S. notomytis* (Eisenberg et al. 2015b), *S. ratti* (Eisenberg et al. 2016) and *S. canis* (Eisenberg et al. 2020b) were described as novel species. Whereas *S. notomytis* and *S. ratti* are closely associated with black rats (*Rattus rattus*), [*S.*] *hongkongensis* has exclusively been isolated from humans (Lau et al. 2016; Woo et al. 2014) and was recently found to belong to a novel genus, *Pseudostreptobacillus* (Eisenberg et al. 2020a). *S. felis* and *S. canis* were only once isolated from clinical disease in animals, i.e. from a cat with pneumonia and a dog with phlegmon, respectively (Eisenberg et al. 2015a, 2020b). However, with respect to zoonotic potential, *S. notomytis* has been found to also cause RBF in humans (Fukushima et al. 2017; Ogawa et al. 2018) and a similar case of RBF could recently be attributed to *S. felis* for the first time (Matt et al. 2020). Interestingly, various *Streptobacillus* phylotypes consistent with 16S rRNA gene sequence based operational taxonomic units (OTU) have been described from humans and various animal species (Fig. 1). We here report a second strain of *S. felis*, isolated from a tropical rusty-spotted cat (*Prionailurus rubiginosus*), one of the smallest members of *Felidae*, that succumbed to infection.

## Materials and methods

### Case description

A breeding group of the endangered rusty-spotted cat (*Prionailurus rubiginosus phillipsi*), a subspecies native to humid zones of Sri Lanka, is managed for ex situ breeding purposes in a German zoo. The cats have been bred in the same zoo or within the European studbook program and are housed individually or in breeding pairs. From the breeding group no significant morbidities and mortalities have occured, but individual animals have suffered from intermittant signs of kitty flu like sneezing, epiphora, elevated respiratory rate, reduced appetite, corneal ulceration in the years before this study. In the actual case, a female displayed bilateral blepharitis, weakness, respiratory distress and anorexia. *Intra vitam* tests for feline parvovirus, coronavirus and protozoa revealed negative results. Due to disease progression, the animal was euthanized.

### Pathological investigation

A gross pathology examination and histology were performed. For histopathological examination, specimens of multiple organs were fixed in buffered 4% formalin, processed by standard methods and embedded in paraffin. Microtome sections were stained with hematoxylin–eosin (HE).

### Immuno-histochemistry (IHC) for *S. moniliformis*

The IHC examination of the formalin fixed paraffin embedded (FFPE) samples taken for histopathological examination was performed using a recently established and not yet published protocol. Briefly, this protocol utilizes a standard IHC procedure with the use of heat induced antigen demasking in target retrieval solution (Dako Cytomation Denmark AS, Glostrup, Denmark), followed by goat serum (Life Technologies Corporation, Paisley, UK) and avidin/biotin blocking agent (Linaris Biologische Produkte GmbH, Dossenheim, Germany) in order to block non-specific binding and reactions, respectively. The primary antibody used was an affinity purified polyclonal rabbit-anti-*S. moniliformis* antibody supplied by Davids Biotechnologie GmbH (Regensburg, Germany). A goat-anti-rabbit IgG biotinylated antibody (Vector Laboratories, Burlingame, USA) served as a secondary antibody and allowed the detection of the antigen–antibody complex using the Vectastain ABC-Elite Kit (Linaris). Diaminobenzidine (DAB; Sigma-Aldrich Chemie GmbH, Steinheim, Germany) was added, resulting in a brown-colored precipitate forming where antibody have bound.

FFPE samples of the lung of a C57BL/6 mouse that was experimentally infected with *S. moniliformis* (Fornefett et al. 2017) underwent the same protocol and served as positive controls. For a negative control, FFPE samples of the rusty-spotted cat underwent the described protocol with only the primary antibody being replaced with negative control rabbit immunoglobulin fraction (Dako). Evaluation of the immune-histochemical examination was performed using a transmission light microscope.

### Phenotypic characterization

#### Bacterial isolation and physiological properties

Bacterial isolates were obtained and isolates were identified using standard microbiological examinations. Briefly, native tissue samples were processed for microbial culture by inoculating flame sterilized, freshly cut tissue surfaces onto culture media (Columbia agar with 5% sheep blood [SBA; Oxoid, Wesel, Germany] and Gassner agar [VWR, Darmstadt, Germany]). Agar plates were incubated for up to 48 h at 20 °C using aerobic and microaerobic culture conditions. Phenotypic characterization of streptobacilli is known to yield only few weakly positive reactions (Eisenberg et al. 2015c), however, standard microbiological procedures included tests for hemolysis on SBA, catalase activity with 3% H_2_O_2_ on microscopic slides and for presence of cytochrome oxidase with the BBL DrySlide^®^ oxidase system (Becton–Dickinson, Heidelberg, Germany). Urease, hydrogen sulfide, indole, motility and oxidative and fermentative glucose assimilation were tested on Christensen agar, SIM and OF medium in slant agar tubes, respectively (all Merck, Darmstadt, Germany). Microscopic examinations of fixed smears were performed using Gram’s stain. For further identification attempts, the Omnilog GEN III plate identification system (Biolog, Hayward, USA) was utilized for the first time using the most sensitive protocols for fastidious bacteria (C1 and C2) with and without addition of 10% bovine serum according to manufacturer’s recommendations.

### Matrix-assisted laser desorption/ionization-time of flight mass spectrometry (MALDI-TOF MS)

Mass spectrometry procedure has been recently described in detail (Eisenberg et al. 2018, 2020b). The commercial database used (DB 8,468; BrukerDaltonics) comprised 24 spectra each from 10 *S. moniliformis* strains. Reference spectra from well-characterized, quality-controlled strains of all other *Streptobacillus*/*Pseudostreptobacillus* species and most other members of the *Leptotrichiaceae* were added to the database from previous studies (Eisenberg et al. 2020b; Rau et al. 2016). Identification was done with the commercial Bruker database, and with the extended database.

#### Molecular characterization of isolate LHL191014123 obtained from liver tissue

##### PCR analysis

Two earlier designed PCR assays for the detection of *S. moniliformis* were employed to detect characteristic amplicon sizes of approximately 269 and 1,190 bp also for the rusty-spotted cat strain LHL191014123 (primers S5: 5′-CAT ACT CGG AAT AAG ATG G-3′ and AS2: 5′-GCT TAG CTC CTC TTT GTA C-3′) (Kimura et al. 2008) and [primers SbmF: 5′-GAG AGA GCT TTG CAT CCT-3′ and SbmR: 5′-GTA ACT TCA GGT GCA ACT-3′; Nicklas, cited in (Rohde et al. 2008)]. It was recently found that these PCR assays are rather genus than species specific (Eisenberg et al. 2015c). Therefore, we have recently designed primers (forward: 5′- AGT ATG GGA AAT AGT AGA TAA TAG-3′ and reverse 5′- ACT GTA GAT TGT GAG TTC TT-3′) that could specifically amplify a partial sequence of the *gyrB* gene (732 bp) of the *S. felis* genome (Matt et al. 2020). The PCR reaction components and cycling conditions were carried out as previously described in Fawzy et al. (2016) with minor modifications (annealing temperature was 53 °C and elongation time was 90 s).

#### Whole genome sequencing

Whole genome sequencing (WGS) was carried out to get insight into a core genome based phylogeny and compare the rusty-spotted cat’s strain with established type strain genomes from the same family. The genome sequence of strain LHL191014123 was generated by de-novo assembly with reads from Illumina technology. In brief, DNA was isolated from cells grown for 3 days at 37 °C on TSA supplemented with 20% horse serum using a PureLink genomic DNA kit (Thermo Fisher). The library was prepared with a Nextera XT library preparation kit (Illumina) and sequenced on NextSeq 500 (mid output kit v2, 2 × 150 bp) instruments. The genome assembly was carried out by SPAdes (version 3.10.1), resulting in 163 contigs with 179 × average coverage.

##### Phylogenetic and phylogenomic analyses

For a first phylogenetic placement, a tree based on nearly full-length 16S rRNA gene sequences was constructed with Geneious vers. 8.1.9 (Kearse et al. 2012) using a Clustal W nucleotide alignment with standard settings and a Neighbor-Joining (NJ) tree (data not shown). Therefore, the 16S rRNA gene sequences of all type strains of the *Leptotrichiaceae* were obtained from GenBank and for strain LHL191014123 deduced from the full genome sequence (s. below). For a more detailed view into the phylogenetic relationship of strain LHL191014123 and all other *Streptobacillus* species the criteria of Woo et al. (2014) were considered. Phylogenetic analyses based on near full-lengths nucleotide sequences of the *groEL*, *gyrB* and *recA* genes were performed for all *Streptobacillus* species and the type species of all other genera of the *Leptotrichiaceae*. Respective nucleotide sequences were aligned using ClustalW implemented in Geneious vers. 8.1.9 (Kearse et al. 2012) and visualized as unweighted pair group method with arithmetic mean (UPGMA) phylogenetic trees (based on 1,000 replications [bootstrap analysis]). A representative tree for the *gyrB* gene is shown in Suppl. Fig. S1. The average nucleotide identity (ANI) values and core genome phylogeny were calculated for strain LHL191014123 in comparison with type strain genomes of the family *Leptotrichiaceae* using the EDGAR 2.3 platform (Blom et al. 2016). ANI values were computed as described by Goris et al. (2007) and as implemented in JSpecies (Richter and Rossello-Mora 2009).

## Results

### Gross pathology

The rusty-spotted cat weighed approx. 1.9 kg, which constitutes a normal weight of this small wildcat. The female was born in 2010 and was 9-years old at the time of death. During *post mortem* examination a light creamy fur, possibly indicating signs of chronic cat flu, was found in the left cavum nasi, accompanied by a light hemorrhagic exudate.

### Histo-pathology

A moderate follicular hyperplasia was noted in the spleen. Focal edema and emphysema were found in histological sections of lung tissue. Focally, fibrin, desquamated alveolar macrophages and neutrophil granulocytes with occasional phagocytized bacteria were detected in bronchioles and pulmonary alveoli. Single cysts were found in the kidneys.

### Immuno-histochemistry

The IHC examination using a method designed for the detection of *S. moniliformis* in tissue samples revealed negative results in all examined tissues of the rusty-spotted cat. Positive and negative controls were successfully showing the expected results, reaffirming sufficient specificity for the detection of *S. moniliformis*.

### General microbiology

Bacterial culture revealed growth of *S. felis* in all tissues, except intestine and intestinal lymph node. The semiquantitative number of streptobacilli as obtained by counting colonies on the directly inoculated agar surface was found to be low (< 20) in spleen and kidney, moderate (20–50) in liver and lung and high (> 50) in the nasal cavity.

Varied growths of other Gram-positive and Gram-negative microbiota were cultivated from other tissues and identified as *Enterococcus faecalis, Vagococcus teuberi* and *Escherichia coli* using MALDI-TOF MS. Selective verification procedures for purely microaerobic bacteria, *Chlamydia* spp., *Mycoplasma* spp. or *Salmonella* spp. and herpesviruses revealed negative results throughout (data not shown).

### Parasitology

A coproscopy flotation technique was negative.

### Phenotypic characterization of isolate LHL191014123 obtained from liver tissue

#### Biochemical identification

Due to the fastidious growth, physiological reaction patterns are generally very weak in members of the genus *Streptobacillus* [Eisenberg et al. 2015c]. The physiological characterization of strain LHL191014123 with the Omnilog GEN III plate identification system did not reveal a superior resolution compared to other standard tests, although a panel of 94 different reactions was assessed. Even the most sensitive protocols for fastidious bacteria (C1 [24 h] and C2 [48 h incubation]) with and without addition of 10% bovine serum resulted in very weak reactions. Slightly positive reactions were found only for l-aspartic acid, l-glutamic acid, D-glucuronic acid, glucuronamide, L-lactic acid, citric acid, α-keto-butyric acid, sodium butyrate and sodium bromate (data not shown).

### MALDI-TOF MS

**Fig. 2:**
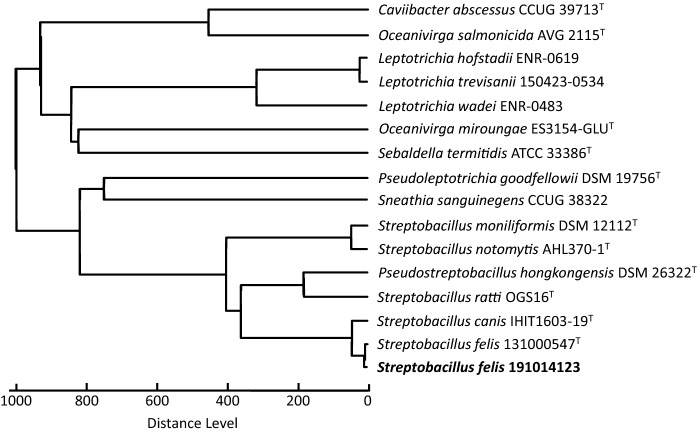
Dendrogram including reference main spectra (MSP) of the family *Leptotrichiaceae* available in the Bruker Taxonomy Database; spectra of *Streptobacillus canis* IHIT1603-19^T^, S. *felis* 131000547^T^, S. *notomytis* AHL 370-1^T^, S. *ratti* OGS16^T^, *Pseudostreptobacillus hongkongensis* DSM26322^T^, *Caviibacter abscessus* CCUG39713^T^, *Oceanivirga salmonicida* AVG2115^T^, *Oceanivirga miroungae* ES3154-GLU^T^, *Sebaldella termitidis* NCTC11300^T^, *Sneathia sanguinegens* CCUG41628^T^ reference strains were recorded using an acetonitrile-formic acid extraction protocol. The dendrogram was generated using the MBT Compass Explorer MSP Dendrogram Creation Standard Method (v1.4) of the MALDI Biotyper OC Software (v3.1, build 66). The database used (DB 8,468, BrukerDaltonics) comprised only strains of *Streptobacillus moniliformis* including the the type strain DSM 12112^T^ as well as spectra of the depicted *Leptotrichia* spp.; ^T^, type strain; ENR, European Network for the Rapid Identification of Anaerobes (ENRIA)

MALDI-TOF MS spectra of strain LHL191014123 show the *m/z* signals typical of *Streptobacillus* genus at 3,631.2 ± 3.6, 7,262.0 ± 7.3, and 7,392.0 ± 7.4 (data not shown, Eisenberg et al. 2018). Nevertheless, the strain LHL191014123 had score values lower than 1.5 with the used commercial Bruker database version and was, therefore, not identified. The application of the enlarged database, extended by reference entries for each of the known members of the genus *Streptobacillus*, including the type strain of *S. felis* 131000547^T^, allowed the unequivocal assignment of strain LHL191014123 to *S. felis* (score values up to 2.719). Custom-made MSP of all *Streptobacillus* species were prepared from known type strains based on Bruker quality criteria and used in this study; respective MSP are available for exchange via the MALDI-user platform MALDI-UP (https://maldi-up.ua-bw.de) (Rau et al. 2016). A dendrogram depicting the topologic position of the reference spectrum of strain LHL191014123 from the rusty-spotted cat from this study to closely related strains of the other known streptobacilli and related taxa is shown in Fig. 2.

## Molecular characterization

### PCR analysis

Both earlier published PCR protocols for the detection of *S. moniliformis* are based on the *Streptobacillus* 16S rRNA gene. As expected, strain LHL191014123 from this study gave a positive amplification in these two PCR assays. The recently designed *gyrB* gene PCR was also positive for strain LHL191014123.

### Genomic features

The draft genome (1,386,907 bp) consists of 163 contigs and possesses 1,345 CDS, 2 rRNA and 38 tRNA. Analysis of further genomic features revealed one prophage (PHAGE_Gordon_Smoothie_NC_030696, (Zhou et al. 2011)) and 96 tandem repeats (Benson 1999). However, screening for CRISPR regions was negative in contrast to *S. felis* type strain (131000547^T^) that was found to possess a relatively large CRISPR region (2,078 bp) with 31 spacers (Grissa et al. 2007). Five genomic islands with a size range 3,220 to 66,556 bp were also identified (Bertelli et al. 2017). Four islands possess mainly hypothetical proteins and none seems to express pathogenic factors. However, one island (26,565 bp) seems to be associated with transport and metabolism of different substrates including carbohydrates and minerals. Interestingly, genome analysis with Pathogenfinder (Cosentino et al. 2013) suggested that *S. felis* (LHL191014123 as well as 131000547^T^) has a probability of 0.976 to be a human pathogen since it harbors seven pathogenic families, all of which originate from *S. moniliformis*, the classical pathogen of the RBF zoonosis. This web-tool predicts the pathogenicity of a submitted genome based on a model that compares its sequence data to a protein family database containing proteins known to be associated with pathogenic or non-pathogenic bacteria.

### Phylogenetic and phylogenomic analyses

**Fig. 3:**
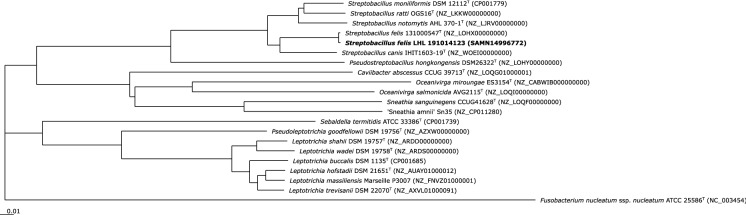
Core genome phylogenetic tree depicting strain LHL191014123 within the family *Leptotrichiaceae*. Core genes of these genomes were computed in EDGAR 2.3 based on MUSCLE alignments and the Neighbor-Joining algorithm as implemented in the PHYLIP package. The core genome analysis was based on of 267 genes per genome in 17 type species genomes (5,607 in total) of the family *Leptotrichiaceae*. The core has 95,146 amino acid residues and 1,998,066 bp per genome in total. GenBank accession numbers are given in parentheses. “Sneathia amnii” and “Leptotrichia massiliensis” were included, however, these taxonomic names have been effectively published but not validly published under the rules of the International Code of Nomenclature of Bacteria. *Fusobacterium nucleatum* is used as outgroup. ^“T”^ indicating type strain; Bar, 0.01 amino acid substitutions per site

The 16S rRNA gene sequence of strain LHL191014123 was derived from WGS and represents a stretch of 1,514 unambiguous nucleotides. This sequence was blasted against the quality-controlled database EzBioCloud (Yoon et al. 2017) and highest similarities to the type strains of *S. felis* (99.93%), *S. canis* (98.68%), *S. notomytis* (98.26%), *S. ratti* (97.85%), *S. moniliformis* (97.64%) and *P. hongkongensis* (94.23%), followed by *Oceanivirga salmonicida* (91.10%) and ‘Sneathia amnii’ (90.58%) were found. In a 16S rRNA gene sequence phylogenetic tree (ML algorithm), strain LHL191014123 clustered most closely and in a separate branch together with the type strain of *S. felis*. The next closely related species was *S. canis* that grouped as a sister clade to *S. felis* with high bootstrap support (data not shown). Based on partial nucleotide sequences of the *groEL*, *gyrB* (Suppl. Fig. S1) and *recA* genes, this topologic position was also identical for the investigated housekeeping genes. A core genome phylogeny of strain LHL191014123 and 20 genomes of the family *Leptotrichiaceae* was calculated in EDGAR 2.3 based on MUSCLE alignment as previously described (Eisenberg et al. 2017b). This resulted in one multiple alignment of 267 core genes per genome (5,607 genes in total), with 95,146 amino acid residues per genome (1,998,066 in total). The Neighbor-Joining algorithm (Fig. 3) as well as the approximately-Maximum Likelihood phylogeny (data not shown) both confirmed the taxonomic position of strain LHL191014123 as a member of *Streptobacillus felis* with *S. canis* being the closest relative, still with a larger phylogenetic distance. Species identity between the rusty-spotted cat’s strain to *S. felis* was once more confirmed by mean average nucleotide identity (ANI) values of 99.32% (reciprocal 99.24), which is clearly above the > 95–96% proposed boundary for identical species (Goris et al. 2007).

### Accession numbers and strain deposition

The GenBank/ENA/DDBJ accession numbers for the 16S rRNA, *groEL*, *gyrB* and *recA* gene sequences of strain LHL191014123 as well as for the complete genome sequence are MN764137, MN793979, MN793980, MN793981 and genome acc. no. (JABMKT000000000; BioSample SAMN14996772; BioProject PRJNA634464), respectively. Further accession numbers of *gyrB* sequences from mouth swabs from cats are MT498840-MT498846 and have been published in Matt et al. (2020). Strain LHL191014123 has been deposited at the German Collection of Microorganisms and Cell Cultures (DSMZ), the Culture Collection of the University Gotenburg (CCUG), the Collection of Institute Pasteur (CIP) and the strain collection of the Hessian State Laboratory (LHL) under identifiers DSM110500, CCUG74119, CIP111794 and LHL191014123.

## Discussion

It is evident from the depicted molecular results and from MALDI-TOF MS analysis that LHL191014123 is an additional strain of *S. felis*. To our knowledge, this is the second available strain with proper species identification and an extended genetic and phenotypic knowledge base. Although we could recently show that approximately 50% of randomly selected, mostly healthy domestic cats harbor *S. felis* (Matt et al. 2020), the isolation of these streptobacilli from cats is a rare exceptional case. A number of studies have indicated dogs and cats as possible vectors of *S. moniliformis* to humans, especially after mouthing wild rats (Gascard et al. 1967; Maynard et al. 1986; Mollaret 1969; Peel 1993; Wouters et al. 2008). However, isolates have not been stored and phylotypes are not available for most of the mentioned studies. Therefore, these microorganisms cannot be verified as *S. moniliformis*. Conversely, 16S rRNA gene phylotypes from one former and additional studies in dogs suggested a much closer relationship of their *Streptobacillus* OTUs to *S. canis* than to *S. moniliformis* (Dewhirst et al. 2012; Xenoulis et al. 2008) (Fig. 1). Relatively few dogs have suffered from streptobacillosis (Das 1986; Ditchfield et al. 1961).

OTU sequences of cats that are most closely related to *S. felis* were previously lacking, but have recently been found in half of the investigated cats (Matt et al. 2020) and also closely related bacterial species (uncultured ‘*Leptotrichia*’, ‘*Leptotrichiaceae*’) have been detected at various body sites (Older et al. 2019; Sturgeon et al. 2014). One study mentions two *Streptobacillus* isolates but without any further identification or disease association (Whyte et al. 2017). The species *S. felis* has been described from cats as well as from a human patient with contact to cats, suggesting that this microorganism may be a member of the cats’ microbiota with the potential to cause zoonotic infections (Eisenberg et al. 2015a; Matt et al. 2020). The high occurrence (50% found in Matt et al. (2020)) should be considered with respect to the potential role of *S. felis* both as a cat pathogen and a potentially zoonotic microorganism because cats represent the most popular pet animal species and streptobacillosis is considered a significantly under-reported disease. However, possible reasons why streptobacilli have been infrequently diagnosed may include a lack of awareness of the disease among clinicians, an absence of pathognomic signs of disease in animals, a lack of reliable diagnostics, fastidious growth of the pathogen, susceptibility to most antibiotics used for empiric therapy, unnoticed animal contact and that this is a non-notifiable disease worldwide (Eisenberg 2017).

*S. felis* has first been isolated from a cat with acute bronchopneumonia and a myocardium with multifocal haemorrhages on the endo- and epicardium (Eisenberg et al. 2014). In this second report, again lungs were predominantly affected, but streptobacilli were isolated from all major organs pointing towards an agonal spread of these bacteria or an early septicemia. Likewise, the next closely related species, *S. canis*, has recently been found to also constitute a member of canine oral microbiota (Matt et al. 2020) and has been isolated from a phlegmon on a dog’s hindleg (Eisenberg et al. 2020b). Hypothesizing that bite wounds are often caused by oral microbiota (Abrahamian and Goldstein 2011), one can speculate that streptobacilli from cats might occasionally also be involved in wound infections.

Interestingly, a novel diagnostic immune-histology tool for the detection of *S. moniliformis* turned out to reveal negative results throughout. The method was found to successfully detect experimentally infected mice (data not shown). Because the lack of suitable diagnostics for the detection of streptobacillosis is often referred to as a diagnostic dilemma (Mahmoodi et al. 2016; Rumley et al. 1987), the novel IHC assay represents a promising diagnostic tool to improve this situation and to identify *S. moniliformis* in situ. The lack of binding in the here presented case suggests deviant epitopes in *S. felis*.

## Conclusion

This is the second isolation of *S. felis* in a diseased cat species. A preliminary sampling of cats revealed that this microorganism is frequently found in the oropharynx and that cats represent a reservoir for *S. felis*. However, this is further evidence that this species is cat specific but with a broader distribution in feline hosts than previously thought. Further studies are necessary to elucidate the role of *S. felis* in domestic and other cat species in order to better estimate its zoonotic potential.

## Electronic supplementary material

Below is the link to the electronic supplementary material.

Fig. S1. Phylogenetic tree based on partial *gyrB* gene (1,983 nt) sequences including type strains of all genera of the family *Leptotrichiaceae* showing the phylogenetic relationship of strain LHL191014123 to other *Streptobacillus* species. The UPGMA consensus tree was generated in Geneious vers. 8.1.9 (Kearse et al. 2012) using a Clustal W nucleotide alignment with standard settings and rapid bootstrap analysis (1,000 bootstraps). GenBank accession numbers are given in parentheses. Numbers at branch nodes refer to bootstrap values; Fusobacterium nucleatum is used as outgroup. ^“T”^ indicating type strain; Bar, 0.06 nucleotide substitutions per site (PDF 394 kb)

## Data Availability

All data have been made fully available to the public.
