# Supplementary figures and images for: Streptobacillus felis, a member of the oropharynx microbiota of the Felidae, isolated from a tropical rusty-spotted cat
Source: Antonie Van Leeuwenhoek. 2020 Aug 9;113(10):1455–65. doi: 10.1007/s10482-020-01454-x (PMC7415334; doi:10.1007/s10482-020-01454-x)

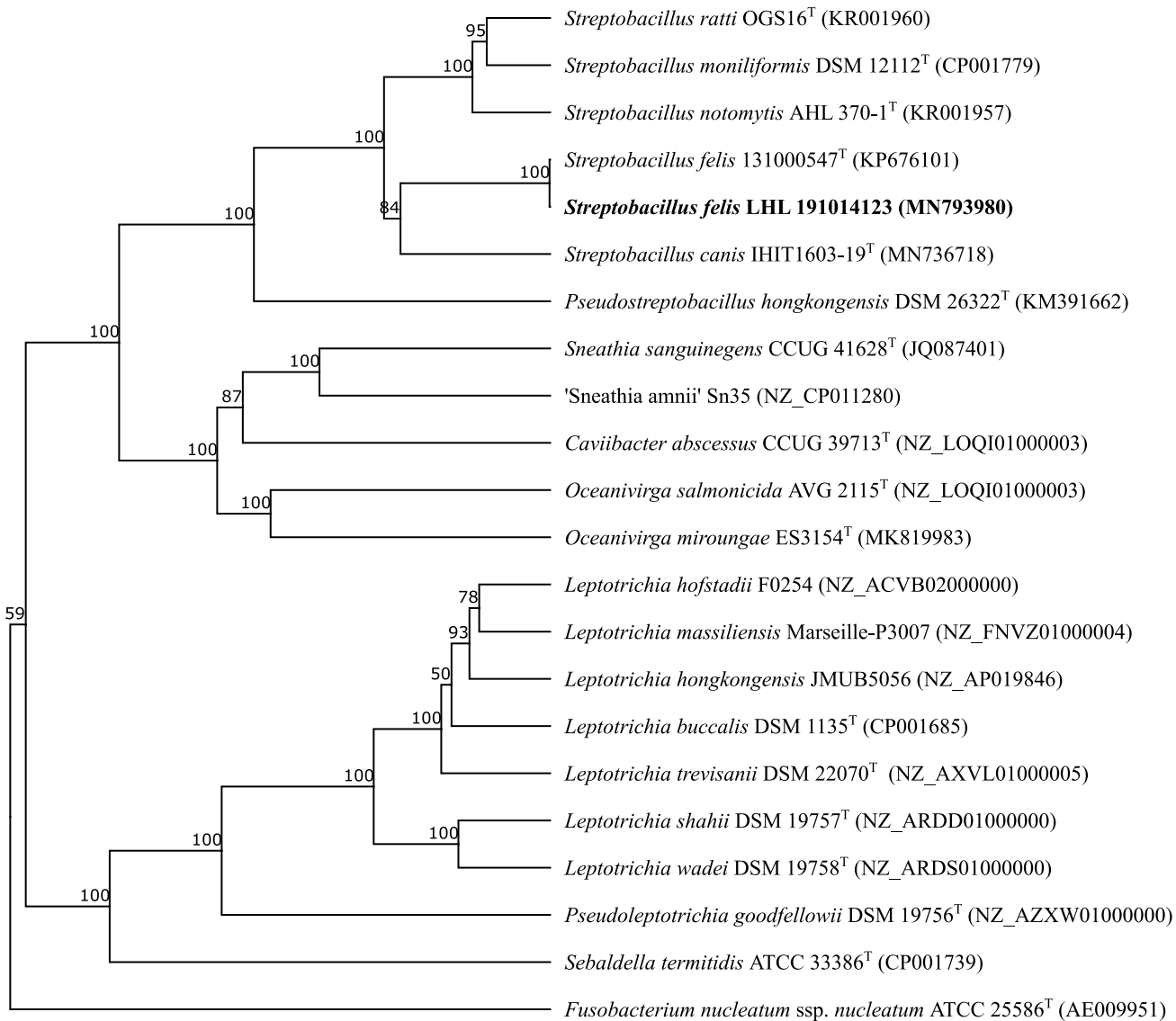

0.06

Supplement: Supplementary file 1 — Fig. S1. Phylogenetic tree based on partial gyrB gene (1,983 nt) sequences including type strains of all genera of the family Leptotrichiaceae showing the phylogenetic relationship of strain LHL191014123 to other Streptobacillus species. The UPGMA consensus tree was generated in Geneious vers. 8.1.9 (Kearse et al. 2012) using a Clustal W nucleotide alignment with standard settings and rapid bootstrap analysis (1,000 bootstraps). GenBank accession numbers are given in parentheses. Numbers at branch nodes refer to bootstrap values; Fusobacterium nucleatum is used as outgroup. “T” indicating type strain; Bar, 0.06 nucleotide substitutions per site (PDF 394 kb) [file 10482_2020_1454_MOESM1_ESM.pdf]
